# Enrichment of Whole Wheat Cocoa Biscuits with Encapsulated Grape Skin Extract

**DOI:** 10.1155/2019/9161840

**Published:** 2019-02-03

**Authors:** Roberta Dordoni, Guillermo Duserm Garrido, Laura Marinoni, Luisa Torri, Maria Piochi, Giorgia Spigno

**Affiliations:** ^1^Department for Sustainable Food Process, DiSTAS, Università Cattolica del Sacro Cuore, Piacenza 29122, Italy; ^2^Centre for Dairy and Fodder Crops Research, CREA, Lodi 26900, Italy; ^3^University of Gastronomic Sciences, Bra (CN) 12060, Italy

## Abstract

Grape pomace is one of the major waste products generated by the wine-making process and it contains seeds and skins still rich in bioactive compounds. Skins can be separated from the seeds and valorised for the recovery of antioxidant extracts with different potential applications. The aim of this study was to evaluate the influence of the addition of an extract obtained from waste grape skins and encapsulated in maltodextrins on the antioxidant properties and sensory acceptability of whole wheat cocoa biscuits. Different levels of enrichment (1.2, 2.3, and 3.5% on dough weight) were tested, and the obtained doughs and biscuits were analysed for total phenols content and antioxidant capacity (based on different radical assays). Extract addition increased the phenolic content (up to 134% increase) and antioxidant capacity (up to 244%) of both doughs and biscuits. Extract containing biscuits were also characterised by a different colour. However, oxidation stability, evaluated at accelerated temperature and oxygen conditions, was not improved by the extract incorporation. Cooking led to an average (not always significant) 16% decrease in phenolic content for both enriched and reference recipes. The enrichment level significantly influenced the sensory acceptability, with identification of two clusters of consumers, with one cluster preferring the biscuits with the highest enrichment level and one cluster preferring the biscuits with the lowest level. The results showed that whole wheat cacao biscuits represent an appropriate food matrix to develop pleasant novel products enriched in phenolic compounds from waste grape skins and likeable for regular biscuits consumers.

## 1. Introduction

Wine production is one of the most important agricultural activities in the world with an estimated 2017 production of 246.7 million of hectolitres [1], with the wine-making process producing three main byproducts: pomace (often referred to as grape marc, a mixture of skins and seeds discarded after either grape pressing or must maceration), stems (or stalks), and lees. Grape pomace and stems have been estimated to account for about 10-20% and 2-8% in weight of the processed material, respectively [2]. These residues pose serious environmental concern due to their huge amounts produced in a restricted period of time and high content in organic matter. However, wine-making byproducts are still rich in bioactive compounds that could be recovered to obtain high value-added products to be recycled into the food chain [3, 4]. In particular, grape pomace is a source of fibres and antioxidant phenolic compounds well known for their potential health beneficial effects [5]. Therefore, recovery of phenolic extracts from grape pomace, through different extraction techniques, such as solvent extraction [6], ultrasounds [7], supercritical fluids [8], ohmic heating [9], microwave heating [10], and enzyme digestion [11], has been widely investigated in literature. The obtained extracts can be used in the development of new food products as functional ingredients [12] or as natural antioxidant additives [13].

Due to their wide consumption all over the world, cereal-based products have been largely studied for functionalization, also with ingredients from wine-making byproducts [14]. Among cereal-based products, biscuits are very popular and present on the market in many formulations and recipes: crumbly biscuits, soft or spongy, dry and light, crisp or refined. Nowadays, the food industry, and the bakery food sector, is looking for new formulas to produce healthier foods that meet the consumers' expectation [15].

Only a few studies exist in literature on the enrichment of biscuits with grape byproducts or related extracts [16–20]: Table 1 presents a comparison of the above cited papers in terms of biscuit recipe, type and enrichment level of grape phenolic extracts, and main results related to cooked products. Moreover, only few works on the use of encapsulated extracts in baked goods are available. For example, Davidov-Pardo and colleagues [16] investigated the addition in cookies of microencapsulated extracts specifically from grape seeds. Furthermore, although the effects of microencapsulation on the physicochemical properties of extracts from grape skins have already been explored [21, 22], studies on application of encapsulated extracts in bakery products are still lacking. In the perspective of an industrial approach, using stable and protected grape skin extract could be a strength to ensure the shelf life of the active material and, if necessary, to mask undesirable taste.

Based on these premises, the aim of this study was to evaluate the influence of the addition of an encapsulated waste grape skin extract on the functional properties and sensory acceptability of biscuits.

## 2. Materials and Methods

### 2.1. Red Grape Skin Extract

Grape pomace of the* Barbera* (red grape) variety was kindly provided by a winery located in Northern Italy and treated as previously reported [12, 23] to separate the skins and obtain a crude ethanol extract. Briefly, the pomace was distributed in thin layers in an air oven and dried at 60°C until a residual moisture content < 5-7% was reached (about 24 h). The skins were separated by hand from the seeds and milled to particle size ≤ 2 mm. For extraction, 125 g of grape skin powder were mixed with 1 L of 60% aqueous ethanol under continuous stirring (3500 rpm, mixer Silver-son, L5M) for 2 h, maintaining the temperature of the mixture at 60°C by means of an electric heating plate. The mixture was then centrifuged (at 2000g for 15 min at room temperature) to recover the supernatant extract. The extract was concentrated 15 times with a rotary evaporator (Büchi Rotavapor R-144) and spray-dried with maltodextrins (Glucidex IT 12 DE, dextrose equivalents, kindly provided by Roquette Italia S.p.A.) at a dosing level of 0.6 molar ratio DE/gallic acid equivalents (GAE, total phenols content of the concentrated extract evaluated according to the Folin assay). Spray-drying operation conditions are those reported in Lavelli et al. [12]: 4 mL/min feeding rate with 13% w/v total solids concentration; 667 L/h drying air flow rate, aspirator rate set at 100%; 150°C inlet air temperature.

The obtained grape skin extract encapsulated into maltodextrins (GSM) was stored in a hermetically sealed plastic container and in desiccator until use and was analysed for dry matter content (oven drying at 105 ± 2°C for 24 h), total phenols content (TPC), and encapsulation efficiency.

### 2.2. Biscuits Production

Looking for a healthy profile, after some preliminary trials, the following biscuit recipe was selected: 1000 g whole soft wheat flour (Barilla, Italy), 300 g white sugar (from sugar beet, Zefiro, Eridania, Italy), 250 mL extra virgin olive oil (100% Italian, Coop, Italy), 240 mL mineral still water (Sant'Anna, Fonti di Vinadio S.p.A., Italy), 50 g bitter cocoa powder (Bellarom, Lidl Italia Srl), and 16 g baking powder (containing disodium diphosphate, sodium hydrogen carbonate and corn starch; Belbake, Lidl Stifting & Co KG). All the ingredients were locally purchased. GSM enriched biscuits were prepared replacing part of sugar with the extract. Three levels of extract addition were tested: 1.2, 2.3, and 3.5% w/w on total ingredients, corresponding to 2.2%, 4.4%, and 6.5% on flour weight. The doughs were analysed for moisture content, TPC and AOC.

Biscuits were made using a home kneading machine (Bonmann KM362CB), rolling and shaping the doughs as round biscuits (5 cm diameter, 0.5 cm thickness) and baking in an electric static oven (Eka, 4xGN1/1) 11 min at 180°C.

Biscuits were ground (Moulinex AD5686) and analysed for dry matter content (oven drying at 105 ± 2°C for 24 h), colour, TPC, AOC, oxidation stability, and sensory acceptability.

### 2.3. Colour

Colorimetric analysis was performed using a Spectro-colorimeter Konica Minolta (Chroma Meter CR-400) to measure the Hunter scale parameters L, a*∗* and b*∗*. Chroma C*∗* and hue H(°) were calculated as reported in (1) and (2), respectively:

(1) C∗ab=a∗2+b∗21/2

(2) H()°ab=tg−1b∗a∗;

### 2.4. Total Phenols Content and Encapsulation Efficiency

The TPC of the GSM and the encapsulation efficiency (EE %) were evaluated, following literature [24]. Briefly, the TPC content was assessed after complete dissolution of the extract into ethanol:acetic acid:water (50:8:42 v:v:v): the dispersion was agitated at room temperature using a Vortex (1 min) and then an ultrasonicator twice for 20 min.

TPC was evaluated according to the Folin assay and expressed as mg of GAE per 100 g of GSM by means of a calibration curve with a gallic acid standard (≥98%, Sigma-Aldrich, Steinheim, Germany) between 100 and 800 mg/L [23]. The surface TPC was assessed as the amount of phenolic compounds that solubilise into ethanol:methanol after only 1 min Vortex at room temperature. The EE % was then calculated according to

(3) $$\begin{array}{c} EE\%=\frac{TPC-SurfaceTPC}{TPC}100 \end{array}$$

TPC of doughs and biscuits was evaluated always according to the Folin assay as reported for GSM and expressed as mg GAE/100 g dry matter, following literature [18] for phenols extraction.

### 2.5. Antioxidant Capacity

The same dough and biscuit extracts TPC were assessed for AOC according to the ABTS assay [23]. AOC was expressed as Trolox® equivalents antioxidant activity (TEAC_ABTS_) by a calibration curve obtained with standard Trolox® ((±)-6-Hydroxy-2,5,7,8-tetramethylchromane-2-carboxylic acid, Sigma-Aldrich) between 0.1 – 2.2 mM. TEAC is the ratio of mM Trolox® to mM phenols in the extract (as GAE) resulting in mol_Trolox®_/mol_GAE_ [25].

Based on the TPC of the dough/biscuit, the specific antioxidant capacities related to dry matter of dough/biscuit (TEAC'_ABTS_) were also calculated as *μ*mol_Trolox®_ABTS_/g_dm_.

AOC of dough and biscuit extracts was assessed also as Oxygen Radical Absorbance Capacity (ORAC) [26] and expressed as TEAC_ORAC_ or as TEAC'_ORAC_.

### 2.6. Oxidation Stability

Oxidation stability was assessed using the Oxidation Test Reactor (VELP Scientifica, Milan, Italy). Ground biscuits (30 g) were distributed homogeneously on the instrument sample holders and test conditions were set at 90°C and 6 bar oxygen pressure. The Induction Period (IP in min) was calculated by the instrument software.

### 2.7. Sensory Evaluation

Sensory evaluations were conducted at the Food and Wine Sensory Laboratory of the University of Gastronomic Sciences (Bra, Italy), to assess the degree of acceptability of both the control and enriched biscuits (within one day from preparation). One hundred four consumers (males=44, females=60; mean age=26; range age: 18-69) were involved in the study, including students and staff of the university. Biscuits were individually presented in transparent plastic container (236 mL capacity), hermetically sealed, and identified with a three-digit code numbers. Samples were served at room temperature (25 ± 1°C) in randomized and balanced order among subjects. The consumers were verbally introduced to the computerised data collection procedure (FIZZ Acquisition software, version 2.46A, Biosystèmes, Courtenon, France). General instructions required subjects to rinse their mouth before the beginning of the test and between samples. Participants firstly observed the biscuits and rated their liking for the appearance. Then, subjects smelled the sample and rated liking for odour. Successively, participants tasted each biscuit and judged liking for taste, flavour, and texture. Finally, the overall liking was evaluated. Liking for all parameters was rated on a 9-point hedonic scale ranging from ‘dislike extremely' (1) to ‘like extremely' (9). A timer on the computer screen required the subjects to respect a rest of 60 seconds between samples. Evaluations were conducted in individual booths under white light. At the end of the tasting session, subjects were asked for their frequency of consumption of biscuits (1=less than once a week, 2=1-3 times a week, 3=4-6 times a week, 5=once a day, 6=more than once a day).

### 2.8. Statistical Analysis

Results are reported as mean values of three replicates with their corresponding standard deviations. Data were subjected to one-way analysis of variance (ANOVA) using SPSS (version 20.0, SPSS Inc., Chicago, IL, USA) statistical software to assess the influence of extract addition and cooking on the dough/biscuit properties. In case of significant difference (p < 0.01), Tukey's post hoc test was applied to discriminate means.

Liking data were separately submitted to two-way mixed ANOVA models (fixed factor: sample; random factor: subject) by performing Fisher's Least Significance Difference (LSD; p < 0.05). Overall liking data of all subjects were submitted to Principal Component Analysis (PCA), to obtain an Internal Preference Map (IPM) for explorative purposes. To better explore consumer's preference for biscuit prototypes, a visual oriented approach, based on the inspection of the loading plot, was used for subject clustering and Y-axis was set as limit between the two consumers' segments. Liking data of each cluster were separately submitted to two-way ANOVA models (fixed factor: sample; random factor: subject), by performing Fisher's LSD (p<0.05). Differences between the two clusters considering the declared frequency of consumption of biscuits were analysed by Pearson chi-square distribution. All sensory data analyses were conducted using the software SYSTAT version 13.1 (Systat Software Inc, San José, USA).

## 3. Results and Discussion

### 3.1. Total Phenols Content and Antioxidant Capacity

The GSM showed a moisture content of 5.35 ± 0.14%, a TPC of 111.23 ± 8.02 mg_GAE_/g and an EE% of 82.81 ± 1.15%. Extract addition did not significantly change the moisture content of the dough (Table 2). This was expected since based on the moisture content of the GSM (5.35%) and of sucrose (0.02 ± 0.004%), the calculated theoretical percent increase of moisture content in the dough due to GSM addition would be minimum 0.06 and maximum 0.18%, which is lower than the standard deviation of the evaluated dry matter content.

On the opposite, the enriched biscuits showed a lower moisture content (with exception of the intermediate enrichment level), suggesting the encapsulated extract enhances water release during cooking and, probably, a final harder consistency as sensorially found by Davidov-Pardo et al. [16] and Pasqualone et al. [17]. An increase in hardness was observed also after addition of grape pomace in bread [27], while Aksoylu et al. [19] evaluated a reduced hardness in biscuits added with defatted grape seed powder. Regarding the water content, literature reports both a reduction due to addition of defatted grape seed powder in biscuits [19] and an increase in water activity due to addition of dried grape pomace in bread [27] or in cookies [20]. However, it must be considered that grape pomace powder is a source of dietary fibres whose structural and chemical characteristics play important role in water uptake. In present work biscuits were prepared with grape skin extract encapsulated into maltodextrins DE12 (GSM) by replacing part of sucrose. As known, sucrose influences the texture because it controls hydration and tends to disperse the protein and starch molecules [28]. On the contrary, less moisture absorption occurs with maltodextrin having lower DE (higher molecular weight). Consequently, replacement of part of sucrose with increasing amount of encapsulated extract could also have influenced water holding and biscuit rheology.

The control dough showed a certain TPC probably due to the use of extra virgin olive oil and cocoa powder. Whole wheat flour may also have contributed [29]. Considering the TPC measured in the control dough and the level of extract addition and the TPC of the extract, the theoretical TPC content of the enriched doughs were calculated and compared with the measured values (Table 2). On an average, the extraction procedure could account only for a 58% of the added grape skin phenols. It may be supposed that during kneading, phenols of the extract bind to dough components, such as proteins, and cannot be completely recovered. However, it cannot be excluded that TPC were oxidized, as polyphenol oxidase enzymes are abundant in whole wheat meals [30].

**Figure 1:**
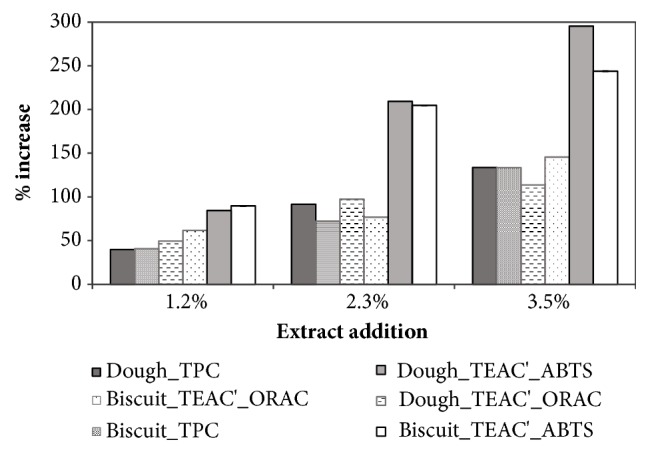
Influence of grape skin extract addition at different levels (1.2, 2.3 and 3.5% on dough weight) on the % increase of total phenols content (TPC) and of specific antioxidant capacity (TEAC' = *μ*mol_Trolox®_/g_dm_ and TEAC = mol_Trolox®_/mol_GAE_) of dough and biscuit, based on the ABTS assay or the ORAC assay. Error bars indicating ± SD are not included since values were calculated from mean values of Table 2 and Figures 2 and 3.

Anyway, GSM addition led to a measured TPC increase in the dough up to 134% for the 3.5% addition level (Figure 1). The % increase of TPC of biscuit with reference to control recipe was almost the same as calculated for the doughs (Figure 1) and in agreement with literature (Table 1) where even higher increases are reported for high replacement ratio of flour with grape skin powder.

Cooking at 180°C led to an apparent average 16.49 ± 4.39% loss in TPC. However, the decrease was statistically significant only for the control biscuit (Sig. 0.035) and the 2.3% extract addition (Sig. 0.002).

Literature is not univocal about the effects of baking on phenolics content which are reported to depend on several factors, such as type of product, type of phenolic compound, recipe, and heating conditions. For instance, an increase in free phenolic compounds due to degradation of conjugated polyphenolic compounds is reported by Abdel-Aal and Rabalski [31] in wholegrain bakery products. Gelinas and McKinnon [32] attributed the increased polyphenols concentration in white bread crust to Maillard reaction products. Stability of anthocyanins after baking was reported by Karakaya et al. [33] in biscuits enriched with commercial grape skin extract. Conversely, Li and collaborators [34] reported significant reduction in total phenolic and total anthocyanins content and ORAC values in purple wheat bran-enriched muffins, but without any influence on the DPPH AOC. Pasqualone et al. [17] observed a decrease of phytocompounds in grape pomace extract fortified biscuits production (some anthocyanins 40% loss), based on the enrichment level and the final measure, without evaluation of the content of the dough. Our findings are in accordance with this latter research, since we observed an apparent loss of the extract phenols of about 40% after kneading and a further not significant reduction after baking. This work, together with a previous study on the use of the same GSM in pasteurised apple puree [12], would suggest a thermal protection given by encapsulation which would, then, be essential in order to exploit active phenolic compounds in thermally processed food products [16, 22].

The addition of GSM also significantly increased the TEAC' of doughs and biscuits with different results depending on the applied assay. In fact, the % increases based on the ORAC results were comparable to those calculated for the TPC, while the ABTS assay gave higher variations (Figure 1).

**Figure 2:**
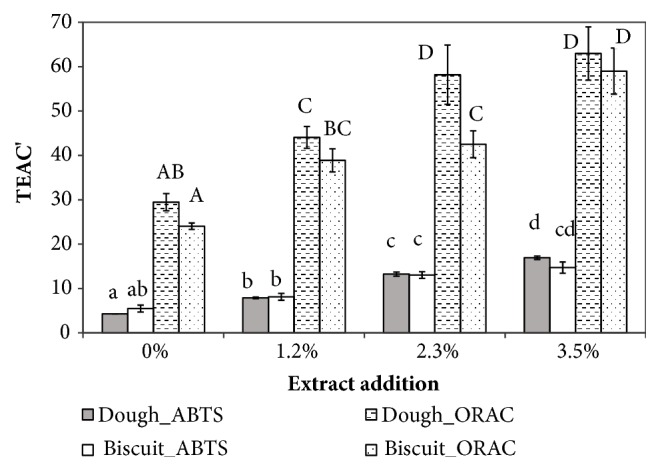
Influence of grape skin extract addition at different levels (0, 1.2, 2.3, and 3.5% on dough weight) on the specific antioxidant capacity (TEAC' = *μ*mol_Trolox®_/g_dm_) of dough and biscuit, based on the ABTS assay or the ORAC assay. Different lowercase letters indicate means statistically different for the ABTS results, while uppercase letters indicate means statistically different for the ORAC results, according to ANOVA analysis and post hoc Tukey's test (p<0.01). Error bars indicate ± SD of mean values (n = 3).

The TEAC'_ABTS_ (Figure 2) significantly increases with extract addition in both dough and biscuit accordingly, while the TEAC'_ORAC_ of doughs with 2.3% of GSM was not statistically different from the 3.5% formulation, and that of 1.2% GSM biscuits was the same as for the 2.3% GSM biscuits. The observed percent increase in TEAC' was in the range of literature data (Table 1) which, however, reports also definitely higher values based on the DDPH test.

Interestingly, both TEAC'_ABTS_ and TEAC'_ORAC_ (with exception for the 2.3% enrichment level) were not significantly influenced by cooking (Figure 2), confirming what was previously commented for the TPC.

**Figure 3:**
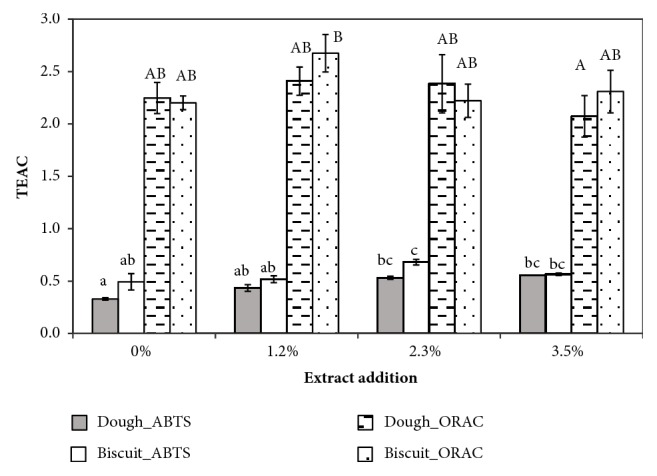
Influence of grape skin extract addition at different levels (0, 1.2, 2.3, and 3.5% on dough weight) on the specific antioxidant capacity (TEAC = mol_Trolox®_/mol_GAE_) of dough and biscuit, based on the ABTS assay or the ORAC assay. Different lowercase letters indicate means statistically different for the ABTS results, while uppercase letters indicate means statistically different for the ORAC results, according to ANOVA analysis and post hoc Tukey's test (p<0.01). Error bars indicate ± SD of mean values (n = 3).

These results suggest a strong correlation between the specific antioxidant capacity and the TPC. In fact, TEAC was almost not significantly influenced neither by the GSM addition level, nor by the cooking process for both the ORAC and the ABTS assay (Figure 3). The found values of TEAC_ABTS_ were slightly lower than those (between 0.5 – 1) found by Spigno et al. [25] for a grape pomace extract.

For comparison purpose, the TEAC for the works cited in Table 1 has been calculated, where possible. The TEAC_ABTS_ of the cookies with red grape pomace extract by Pasqualone et al. [17] was lower than that of our biscuits but, similarly, slightly higher for the enriched biscuits. The TEAC_DPPH_ values calculated from the data of Mildner-Szkudlarz et al. [18] were higher than the TEAC_ABTS_ or TEAC_ORAC_, reflecting the corresponding TEAC', but they confirmed the limited variation of this parameter with grape pomace enrichment.

### 3.2. Colour and Oxidation Stability

Food colour is one of the main factors for consumers' acceptability. The impact of the addition of GSM on biscuit colour was then evaluated (Table 3). The addition of GSM led to a colour variation of control biscuit (see Figure S1 in the Supplementary Material). The coordinate (L) decreased with extract addition without significant increase from 2.3 to 3.5%. The red component of the colour (a*∗*) was significantly reduced by extract addition without influence of the enrichment level. Also, the value of (b*∗*) statistically decreased after extract addition with, in this case, a dose-dependent relation. Similarly, the hue (H(°)) and the colour saturation (C*∗*) significantly decreased with increasing percentage of extract addition. A high saturation value indicates more vivid and bright colours, while high values of H(°) are related to red tones. Similar results were reported by Hayta et al. [27] for red grape pomace enriched bread. However, more often, literature reports an increase in a*∗* following addition of red grape extracts or powder (Table 1). The presence of cocoa in our formulation may have affected and partly masked some colour tones in enriched biscuits.

Oxidation stability tests were performed to evaluate the efficacy of GSM as a natural antioxidant against lipid oxidation in biscuits. The results, in terms of IP, are shown in Table 3. Extract addition did not statistically influence the oxidative stability of our biscuit recipe. Trials carried out with the same extract but encapsulated with different carrier materials showed a protective effect against oxidation of hazelnut paste [13]. Encapsulation formulation and food matrix of application may, then, play a fundamental role in the antioxidant role of the extract.

### 3.3. Sensory Acceptability

Results from the 2-way mixed ANOVA model showed a significant effect of product on liking for texture (F=2.776, p<0.001). This could be explained with the observed results of lower moisture content (except for the intermediate enrichment level) which might have modified the consistency. Despite differences found in colorimetric parameters (Table 3), no significant effect of the product was found for liking of the appearance (Table 4). Moreover, no effect was found for liking of the odour, the taste, and the flavour and the overall liking (Table 4). Liking for texture was significantly higher for biscuits with the highest and lowest GSM addition level than for the control sample. In general, overall mean values correspond approximately to the judgement “5=neither dislike nor like”, which is the central value of the hedonic scale used. This result could be partially due to the tendency of untrained subjects (as the consumers are) to use the middle section of a category scale [35]. Also, Davidov-Pardo et al. [16] reported that overall consumers equally rated control cookies and cookies enriched with microencapsulated grape seed extract, providing average values close to 5.5.

**Figure 4:**
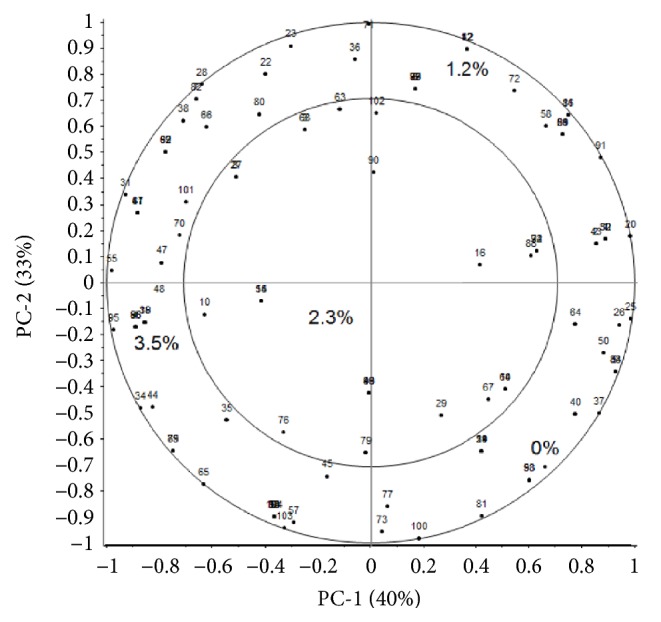
Internal Preference Map of consumers (n=104) who rated the overall liking for biscuits enriched with encapsulated grape skin extract at different levels (0, 1.2, 2.3, and 3.5% on dough weight).

The IPM obtained from PCA applied to the overall liking data is reported in Figure 4. The total variance explained based on the two first significant dimensions was 73%. Samples were spread over the whole space, indicating a neat sensory variability among the enriched biscuits. Samples were distributed according to the increasing level addition of GSM and negative correlation with PC1 gradually increased from 0 to 3.5% of addition. The map revealed that the similar overall liking average values were due to a large variability of the hedonic scores among subjects. In fact, a uniform distribution of consumers all over the space was observed, indicating that preferences of participants for the samples were very variable. From visual segmentation, two clusters showing opposite preferences were obtained: Cluster 1 (Cl1= 53% of subjects, 24 males, 31 females) included subjects negatively correlated to PC1 and preferring the samples with the highest levels of GSM addition, while Cluster 2 (Cl2= 47% of subjects, 20 males, 29 females) included subjects positively correlated to PC1 and preferring the biscuits with the lowest levels of addition. Results of ANOVA models (Table 4) revealed a significant effect of product on liking both for Cl1 and Cl2, except for appearance and odour in Cl1.

For this cluster, even if the colour of the samples was evidently different, the visual aspect of the cookies did not affect neither the liking for appearance nor the overall liking, confirming the findings of Pasqualone et al. [17] on biscuits made with grape pomace extract. Cl1 significantly preferred the samples with the highest levels of addition in terms of overall liking and liking for taste and texture. On the contrary, Cl2 clearly preferred plain standard biscuit, which resulted “neither dislike nor like” both for appearance and overall liking. Considering the appearance, Cl2 significantly disliked the biscuits with the highest GSM addition, compared to the other enriched samples, which were not significantly discriminated. By Cl2, taste, flavour, and overall liking significantly decreased as the level of addition increased, as reported on biscuits enriched with white grape pomace [18]. The opposite preferences of the two clusters are coherent with the preference test results reported by Pasqualone and colleagues [36] showing that a significant difference between the number of consumers who preferred enriched biscuits and the number of subjects who preferred the control biscuits was not found.

A significant difference was observed between the two clusters in terms of frequency of biscuits consumption (Pearson *χ*2= 8.479; p=0.076). Cl1 comprises a higher proportion (36%) of strong biscuits consumers (declaring to consume biscuits “once a day” or “more than once a day”) compared to Cl2 (14%). Interestingly, the Cl who preferred the enriched biscuits is the most numerous one and the one composed by the highest percentage of consumers with a high frequency of biscuits consumption.

## 4. Conclusions

The addition of a red grape skin encapsulated extract in the formulation of a whole wheat cocoa biscuit had positive effects in terms of increase of both total phenols content and antioxidant capacity (according to different assays) of the final product. An apparent loss of almost 40% of the phenols added with the extract was already observed with the analysis of the dough, probably due to phenols binding with ingredients or to oxidation phenomena occurring during kneading. The phenols reduction due to baking was about 16% but not always significant. In the same way, the antioxidant activity of the product was not influenced by cooking. The results, together with comparison with literature, suggest encapsulation might have thermally protected the bioactive compounds during the process.

Extract addition significantly modified the baked product colour, without being detrimental for consumers' acceptability.

Results of consumer's test confirmed that the use of GSM to develop enriched biscuits is a promising issue. The significant preference observed for the cluster including the strongest biscuit consumers for the samples made with the highest GSM levels seems to indicate that whole wheat cacao biscuits represented an appropriate food matrix to develop pleasant novel products likeable for regular biscuits consumers.

## Figures and Tables

**Table 1:** Comparison of literature works on the enrichment of biscuits with grape phenolic extracts or powder from grape byproducts. TPC: total phenols content; GAE: gallic acid equivalents; dm: dry matter; TEAC': trolox equivalent antioxidant capacity (*µ*mol_Trolox_/g) based on ABTS, ORAC, or DPPH radical assay; FRAP: ferric reducing activity power; ↑: increase; ↓: decrease; *↔* constant; IC_50DPPH_ the concentration required to obtain 50% DPPH radical scavenging.

**Biscuit recipe**	**Phenolic extract**	**Enrichment level**	**Main results related to final cooked product**	**Reference**
Whole wheat flour (100 g), sucrose (30 g), extra-virgin olive oil (25 mL), bitter cocoa powder (5 g), water (24 mL), baking powder (1.6 g)	Ethanol red grape skin extract encapsulated into maltodextrins, TPC 111.23 mg_GAE_/g	1.2 - 2.3 - 3.5% on dough weight, corresponding to 2.2%, 4.4% and 6.5% on flour weight, and 245, 489 and 723 mg_GAE_/100 g_flour_	TEAC'_ABTS_ from 5.49 (control) to 8.13 – 13.05 – 14.79 (corresponding to 90 – 205 -244% ↑). TEAC'_ORAC_ from 24 (control) to 39 – 42 – 59 (corresponding to 62 – 77 -145% ↑). TEAC_ABTS_ from 0.49 (control) to 0.52 - 0.68 - 0.57 (no significant ↑). TEAC_ORAC_ from 2.20 (control) to 2.67 – 2.22 - 2.31 (no significant ↑). TPC (mg_GAE_/g_dm_) from 1.89 (control) to 2.67 – 3.26 – 4.43 (corresponding to 42 – 72 - 134% ↑). 40% of extract phenols not extractable from the dough. No influence of extract addition on oxidative stability. No influence of cooking on TPC and TEAC. ↓ in L, a*∗*, b*∗*, H(°), C*∗*. Consumer test (104 subjects): no effect on overall liking. Two clusters of subjects with opposite preferences: liking ↑ for Cluster 1 and ↓ for Cluster 2 as the GSM addition level ↑.	Present work

Flour (100 g), sugar (34.5 g), shortening (16.4 g), lecithin (1.4 g), baking powder (1.1 g), salt (0.4 g), water (32.73 g) for the control or (40 g) for enriched cookies.	Commercial GSE 95% phenols as such (bulk extract) or encapsulated by spray-drying with mesquite gum (44%) and zein (56%) (EEG), or with maltodextrin (34%) and zein (66%) (EEM) TPC of encapsulated extract not specified.	1.67% (on flour weight) of bulk GSE or 5.1% of EEG, 5.05% of EEM. Addition corresponding to theoretical 1.2% GAE on biscuit weight.	TEAC'_DPPH_ (referred to dm of biscuit extract) from 0.8 to 6.8 – 7.5 – 7.8 (corresponding to 750 – 837 -875% ↑), showing a thermal protection by encapsulation. ↓ in L, ↑ in a*∗*, b*∗*. Quantitative descriptive analysis QDA® (11 selected panellists): darker biscuits (with more homogenous colour with EEG and EEM); < sweetness perception; aromas and flavours related to whole flour; > cardboard flavour; > dry mouth sensation and < tenderness. Affective sensory evaluation (126 untrained panellists): same preference for control cookie and enriched with EEM.	Davidov-Pardo et al. [16]

Semolina (100 g), sugar (35 g), extra-virgin olive oil (16 mL), water 25 mL (control) or 10 mL (enriched cookies)	Ethanol red grape pomace extract TPC: 2126 mg_GAE_/L	45 mL of extract per 100 g wheat flour, corresponding to 96 mg_GAE_/100 g_flour_	TPC (mg_GAE_/g) from 0.44 to 0.63 mg_GAE_/g (corresponding to 43% ↑). TEAC'_ABTS_ from 0.47 to 0.79 (corresponding to 68% ↑). TEAC_ABTS_ from 0.18 to 0.21. ↑ in L, a*∗*, ↓ in b*∗*. 40% ↓ in anthocyanins after kneading and cooking. QDA® (8 panellists): more intense colour, fruity odour, sour taste but < friability.	Pasqualone et al. [17]

Recipe as AACC method 54-21.	Freeze dried white grape skins (from pomace). TPC: 31.22 mg_GAE_/g_dm_	10 - 20 - 30% replacement of flour weight	TPC (mg_GAE_/g_dm_) ↑ from 0.85 to 2.11 – 3.34 – 4.45 (corresponding to 148 – 293- 423% ↑). TEAC'_DPPH_ from 1270 to 3390 – 5120 – 7550 (corresponding to 167 – 303 -494% ↑). TEAC_DPPH_ from 254 to 273 – 261 – 289. ↑ biscuit diameter, ↓ biscuit thickness, ↓ L, a*∗*, ↑ b*∗*. Maximum 10% enrichment for sensorial acceptance.	Mildner-Szkudlarz et al. [18]

Wheat flour (100 g), fructose (9.21 g), sugar (20.25 g), shortening (11.87 g), NaHCO_3_ (0.4 g), salt (0.69 g),water (16 g), fortification agent (8 g, only for enriched biscuits)	Defatted grape seed powder TPC: not reported	8% replacement of flour weight	↑ TPC (mg_GAE_/g_dm_) from 8.12 to 17.90 (corresponding to 120% ↑), ↓ peroxide value, ↑ antioxidant capacity (as % AOC_DPPH_) from 1.16 to 43.87%. ↑ diameter, *↔* thickness, ↓moisture content, ↓ L, b*∗*, ↑ a*∗*, ↓ hardness. Sensory analysis (10 trained panellists): ↓ flavour score.	Aksoylu et al. [19]

Refined wheat flour, skimmed milk powder, sugar, margarine, baking powder, butter (amount not reported)	Wine grape pomace powder from Cabernet Sauvignon. TPC: not reported	5 – 10 – 15 - 20% replacement of flour weight	↑TPC (mg_GAE_/g) from 0.041 (control) to 0.095 - 0.213 - 0.305 – 0.460 (corresponding to 132 - 413 - 644 - 1022% ↑). ↑ antioxidant activity as FRAP (mg/g) from 4.625 (control) to 11.651 – 29.669 – 51.862 - 75.976 (corresponding to 152 – 541 – 1021 - 1542% ↑). ↑anthocyanins (mg/g) from 0.163 (control) to 2.033 – 2.864 – 3.311 – 3.512 (corresponding to 1147 – 1657 – 1931 – 2055% ↑). ↑flavonoids (mg CTE/g) from 0.320 (control) to 0.627 – 0.887 – 1.347 – 1.133 (corresponding to 96 – 177 – 321 – 254% ↑). ↑tannins (TAE mg/g) from 0.160 (control) to 0.213 – 0.720 – 1.171 – 1.766 (corresponding to 33 – 350 – 632 – 1004% ↑). ↑ water absorbing water, ↑ ash, ↑ colour intensity. Sensory analysis (40 panellists): maximum score to 5% enrichment.	Maner et al.[20]

**Table 2:** Dry matter and total phenols content (TPC) of doughs and biscuits at different levels of grape skin extract encapsulated into maltodextrins (GSM) enrichment. Values are means ± SD (n = 3). The same superscript letter under each column indicates the means are not statistically different according to analysis of variance (ANOVA) and Tukey's post hoc test (p < 0.01). *∗* TPC theoretical value based on the TPC of control dough, extract addition level and extract TPC. GAE: gallic acid equivalents.

GSM addition [% on dough weight]	Dry matter [%]		TPC [mg_GAE_/100 g_dm_]		
	Dough	Biscuit	Dough	Dough Theoretical*∗*	Biscuit
0	80.06 ± 0.59^a^	88.45 ± 0.05^a^	221.52 ± 8.21^a^		189.53 ± 2.79^a^
1.2	79.50 ± 0.11^a^	91.76 ± 0.21^b^	309.80 ± 14.97^b^	385.49	266.51 ± 8.50^ab^
2.3	78.90 ± 0.22^a^	89.58 ± 0.28^a^	424.16 ± 2.55^c^	551.96	326.33 ± 5.90^b^
3.5	78.94 ± 0.12^a^	92.70 ± 0.13^b^	517.70 ± 11.8^d^	715.29	442.66 ± 28.58^c^

**Table 3:** Influence of the addition of encapsulated grape skin extract at different levels on colorimetric parameters and induction period (IP) of biscuits. Values are means ± SD (n = 3). The same superscript letter under each parameter indicates the means are not statistically different according to analysis of variance (ANOVA) and Tukey's post hoc test (p < 0.01).

Grape skin extract addition						IP
[% on dough weight]	L	a*∗*	b*∗*	H(°)	C*∗*	[min]
**0**	39.83 ± 0.29^a^	10.95 ± 0.19^a^	15.63 ± 0.37^a^	61.08 ± 0.47^a^	19.08 ± 0.39^a^	2325.5 ± 184.6^a^
**1.2**	34.84 ± 1.02^b^	7.45 ± 0.82^b^	10.43 ± 0.36^b^	60.57 ± 4.29^a^	12.83 ± 0.25^b^	2020.0 ± 277.2^a^
**2.3**	30.30 ± 0.83^c^	7.53 ± 0.10^b^	6.76 ± 0.23^c^	46.53 ± 0.67^b^	10.12 ± 0.23^c^	1953.5 ± 58.7^a^
**3.5**	28.99 ± 0.53^c^	7.41 ± 0.38^b^	5.42 ± 0.33^d^	40.18 ± 1.30^b^	9.18 ± 0.46^c^	2038.0 ± 4.2^a^

**Table 4:** Influence of the addition of encapsulated grape skin extract at different levels (0, 1.2, 2.3, and 3.5% on dough weight) on biscuit consumers' preference (liking for appearance, odour, taste, flavour, texture, overall liking). Values are means ± SD. The same superscript letter in the same raw indicates means are not statistically different according to analysis of variance (ANOVA) and post hoc Fishers' least significant test (p <0.05).

	Extract addition				F	p
	0%	1.2%	2.3%	3.5%		
Total (n=104)						
appearance	5.07 ± 0.17	5.21 ± 0.17	5.17 ± 0.17	5.00 ± 0.19	0.932	0.425
odour	4.32 ± 0.17	4.20 ± 0.15	4.33 ± 0.17	4.24 ± 0.17	0.311	0.818
taste	4.34 ± 0.18	4.31 ± 0.17	4.54 ± 0.18	4.38 ± 0.18	0.743	0.527
flavour	4.29 ± 0.17	4.58 ± 0.17	4.52 ± 0.19	4.38 ± 0.18	1.334	0.263
texture	4.13 ± 0.20^b^	4.51 ± 0.20^a^	4.47 ± 0.21^ab^	4.66 ± 0.20^a^	2.776	0.041
overall liking	4.50 ± 0.17	4.60 ± 0.16	4.72 ± 0.16	4.59 ± 0.17	0.659	0.578

Cluster 1 (n=55)						
appearance	4.96 ± 0.26	5.33 ± 0.24	5.29 ± 0.26	5.35 ± 0.26	1.957	0.123
odour	4.00 ± 0.23	4.13 ± 0.21	4.35 ± 0.27	4.51 ± 0.24	2.227	0.087
taste	3.87 ± 0.24^b^	4.11 ± 0.25^b^	4.76 ± 0.26^a^	5.07 ± 0.24^a^	12.599	0.001
flavour	3.95 ± 0.23^b^	4.60 ± 0.24^a^	4.82 ± 0.27^a^	4.93 ± 0.24^a^	8.505	0.001
texture	3.78 ± 0.29^c^	4.47 ± 0.30^b^	4.85 ± 0.32^ab^	5.22 ± 0.25^a^	8.698	0.001
overall liking	4.04 ± 0.22^c^	4.42 ± 0.22^c^	4.91 ± 0.25^b^	5.35 ± 0.23^a^	15.134	0.001

Cluster 2 (n=49)						
appearance	5.18 ± 0.22^a^	5.08 ± 0.23^a^	5.04 ± 0.22^a^	4.61 ± 0.27^b^	2.733	0.05
odour	4.67 ± 0.24^a^	4.29 ± 0.22^ab^	4.31 ± 0.21^ab^	3.94 ± 0.22^b^	4.153	0.001
taste	4.51 ± 0.28^a^	4.55 ± 0.26^ab^	4.04 ± 0.27^b^	4.04 ± 0.28^c^	13.329	0.001
flavour	4.67 ± 0.25^a^	4.55 ± 0.24^ab^	4.18 ± 0.25^b^	3.76 ± 0.26^c^	7.301	0.001
texture	4.86 ± 0.25^a^	4.53 ± 0.24^a^	4.29 ± 0.26^b^	3.61 ± 0.24^b^	3.684	0.05
overall liking	5.02 ± 0.22^a^	4.80 ± 0.23^ab^	4.51 ± 0.20^b^	3.73 ± 0.21^c^	19.286	0.001

## Data Availability

The data used to support the findings of this study are available from the corresponding author upon request.

## Supplementary Materials

Supplementary Materials The appearance of the whole wheat cocoa biscuits is shown in the Supplementary Material (Figure S1).
