# Supplementary material for: Enrichment of Whole Wheat Cocoa Biscuits with Encapsulated Grape Skin Extract
Source: Int J Food Sci. 2019 Feb 3;2019:9161840. doi: 10.1155/2019/9161840 (PMC6378017; doi:10.1155/2019/9161840)

Figure S1: The appearance of the whole wheat cocoa biscuits; control, 1.2 % GSM, 2.3 % GSM, and 3.5 % GSM represent biscuits prepared by adding 0, 1.2, 2.3, and 3.5% of maltodextrin encapsulated grape skin extract (GSM) on dough weight.


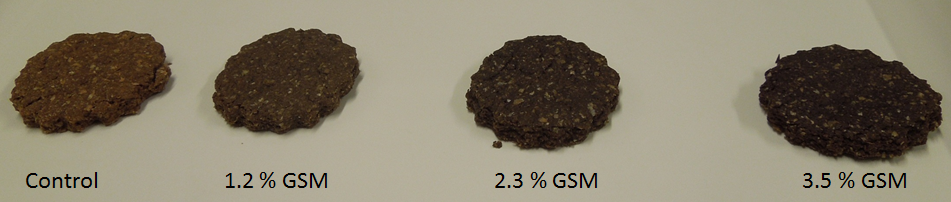

Supplement: Supplementary Materials — The appearance of the whole wheat cocoa biscuits is shown in the Supplementary Material (Figure S1). [file 9161840.f1.docx]
